# Effects of Modified Messenger RNA of Adiponectin Delivered by Lipid Nanoparticles on Adipogenesis and Bone Metabolism In Vitro and In Vivo

**DOI:** 10.3390/cells14120891

**Published:** 2025-06-13

**Authors:** Ying Xie, Qian Ma, Jinghao Wang, Zoe Xiaofang Zhu, Rady E. El-Araby, Maxwell Tu, Zhongyu Li, Xiaoyang Xu, Qisheng Tu, Jake Chen

**Affiliations:** 1 Department of Periodontology, Peking University School and Hospital of Stomatology & National Center for Stomatology & National Clinical Research Center for Oral Diseases & National Engineering Research Center of Oral Biomaterials and Digital Medical Devices, Beijing 100081, China; xieyingpku@163.com; 2 Division of Oral Biology, Department of Periodontology, Tufts University School of Dental Medicine, Boston, MA 02111, USA; maqian@njmu.edu.cn (Q.M.); jinghao_wang@hsdm.harvard.edu (J.W.); zoe.zhu@tufts.edu (Z.X.Z.); rady.el_araby@tufts.edu (R.E.E.-A.); 3 Department of Basic & Clinical Translational Sciences, Tufts University School of Dental Medicine, Boston, MA 02111, USA; maxwell.tu@tufts.edu; 4 Department of Chemical and Materials Engineering, New Jersey Institute of Technology, Newark, NJ 07102, USA; zl396@njit.edu (Z.L.); xiaoyang.xu@njit.edu (X.X.); 5 Department of Genetics, Molecular and Cell Biology, Tufts School of Graduate Biomedical Sciences, Tufts University School of Medicine, Boston, MA 02111, USA

**Keywords:** modified mRNA, adiponectin, adipogenesis, osteogenesis, lipid nanoparticles

## Abstract

Adiponectin (APN) is a secreted adipokine that plays a key role in modulating energy and bone metabolism, as well as regulating inflammatory responses. The overexpression of APN has been proposed as a potential therapeutic strategy for treating obesity and related disorders. Lipid nanoparticles (LNPs) are promising vectors for transporting messenger ribonucleic acid (mRNA) molecules. This study tested whether delivering a stabilized version of adiponectin mRNA (APN mRNA) using lipid nanoparticles could reduce fat formation and promote bone repair in vitro and in vivo. We demonstrated that transfection with APN-LNP upregulated the mRNA and protein expression of APN, while inhibiting adipogenesis in 3T3-L1 adipocytes. APN-LNP enhanced osteogenic gene expression in MC3T3-E1 cells in a dose-dependent manner. It also reduced matrix metalloproteinase 9 expression in receptor activator of nuclear factor-kappaB ligand (RANKL)-stimulated RAW264.7 cells, suggesting an anti-resorptive effect. In vivo, a femoral fracture model was established to explore the application of APN-LNP in promoting bone healing in diet-induced obese mice. Micro-computed tomography and histology analysis indicated that intravenous injection with APN-LNP promoted bone healing. Fasting blood glucose and body weight were decreased in the APN-LNP group. Moreover, APN-LNP increased bone sialoprotein and runt-related transcription factor 2 expression in contralateral femurs, as well as interleukin-10 expression in white adipose tissues. Thus, our study provides promising preclinical data on the potential use of APN-LNP for treating bone disorders in obesity.

## 1. Introduction

Obesity is a chronic disease characterized by excessive accumulation of body fat and is a major risk factor for many diseases, among them type 2 diabetes, cardiovascular disease, and bone disorders [1,2,3]. It is associated with elevated levels of pro-inflammatory cytokines, leading to chronic inflammation, which inhibits osteogenesis and promotes osteoclastogenesis, ultimately delaying bone healing during the bone remodeling process after bone injury [4,5,6,7,8]. Moreover, obesity can alter the production of hormones that regulate bone metabolism, such as adiponectin (APN) and leptin [9]. Excessive mechanical stress on bones due to obesity may also increase the risk of fractures [8,10].

APN is an adipokine (hormone secreted by fat cells) that plays a key role in regulating metabolism and inflammation [11,12]. It can inhibit the expression of pro-inflammatory cytokines associated with obesity [13,14,15]. APN levels are typically decreased in obese individuals [16,17]. Studies have shown that APN can promote osteogenesis and inhibit osteoclastogenesis, suggesting that it may be a potential therapeutic target for obesity-related bone disorders. APN suppresses osteoclastogenesis by activating APPL1-mediated inhibition of Akt1 signaling [18,19]. It also promotes osteoblast differentiation through Runx2 and BMP2 activation [20].

Protein therapy is a traditional approach to treating diseases that involves delivering proteins into the body. However, protein therapy has several limitations. First, it requires high doses of protein, which can be expensive and have safety concerns. Second, proteins are often rapidly degraded in the body, which means that they need to be administered frequently. Messenger RNA (mRNA) therapy is a new and promising approach to treating diseases [21,22,23]. It involves delivering mRNA into cells, where it can be translated into proteins. mRNA therapy has several advantages over traditional protein therapy, including higher efficiency, lower toxicity, and greater specificity.

Compared with DNA therapy, it does not cause changes in DNA modification and mutation. Although mRNA has the potential disadvantages of instability and high cytotoxicity, scientists have made a lot of efforts to improve the stability and reduce cytotoxicity through modifications of the structure of mRNA [24,25,26].

Multiple delivery systems, such as lentiviral vectors, adeno-associated virus, and exosomes, have been developed to transport mRNA into the body [27]. Among these, lipid nanoparticles (LNPs) are a type of nanoparticle that can encapsulate and protect mRNA molecules from rapid clearance by the body by effectively delivering mRNA into cells [28,29,30]. LNPs are non-viral vectors, which means that they do not insert their DNA into the host genome. LNPs are also biocompatible, have low immunogenicity and cytotoxicity, and can effectively deliver mRNA to a variety of tissues [25,31].

Based on these advancements, we chemically modified the APN mRNA and encapsulated it within LNPs. This study aimed to explore the effects of LNPs delivered modified APN mRNA on adipogenesis and bone metabolism in vitro and in a femoral fracture model of male mice with diet-induced obesity (DIO).

## 2. Materials and Methods

### 2.1. Synthesis of APN-LNP

**Figure 1:**
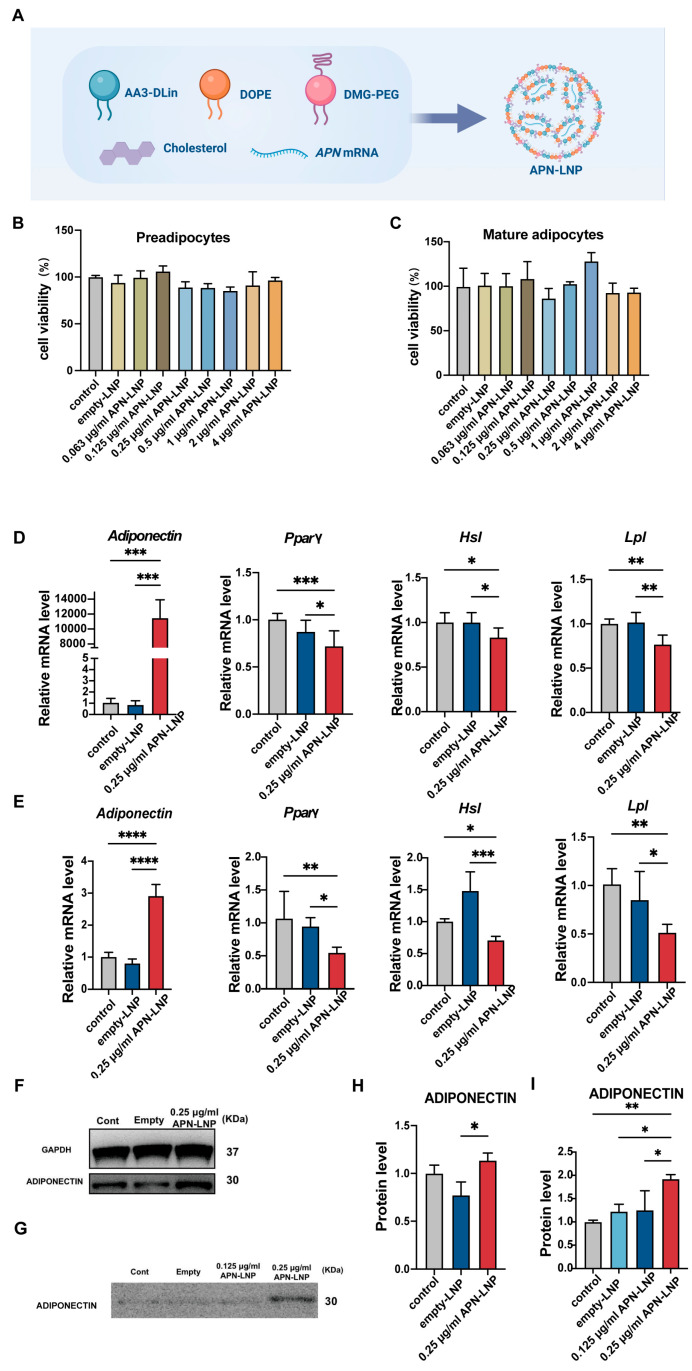
APN-LNP transfection enhances adiponectin (APN) expression and suppresses adipogenesis-related markers at different stages of 3T3-L1 cell differentiation. (**A**) Schematic overview of the synthesis of APN-LNP. The cytotoxicity of APN-LNP in preadipocytes (**B**) and mature adipocytes (**C**) was assessed using the CCK8 assay after 24 h of transfection. Preadipocytes and mature adipocytes were transfected with APN-LNP (0.25 μg/mL) for 24 h, with empty-LNP serving as the control. The mRNA levels of APN and adipogenesis-associated markers, including *Pparγ*, *Hsl*, and *Lpl*, were quantified by qPCR and normalized to GAPDH expression in preadipocytes (**D**) and mature adipocytes (**E**). APN protein levels in cellular fractions (**F**) and the culture medium (**G**) of mature adipocytes following 24 h APN-LNP transfection were analyzed by western blot. Quantitative densitometric analysis was conducted for protein expression in cellular fractions (**H**) and the culture medium (**I**). Data are expressed as mean ± SD. * *p* < 0.05, ** *p* < 0.01, *** *p* < 0.001 and **** *p* < 0.0001. Data were expressed as the mean ± SD at three biological independent experiments.

We chemically modified the APN messenger RNA (cmRNA). Briefly, cmRNA encoding APN was generated by in vitro transcription using T7 RNA polymerase. APN full-length mRNA (NM_009605) was synthesized (BioSynthesis, Inc., Lewisville, TX, USA). To decrease anti-RNA immune response and enhance RNA stability, the mRNA was chemically modified using ribonucleotide substitution, in which 5-Methylcylidine substitutes cytidine residues and pseudouridine substitutes uridine residues. Additionally, a 7-methylguanylate cap was incorporated at the 5′ end, and a poly(A) tail, extended to 120 nucleotides, was added at the 3′ end to ensure efficient translation in target cells. The quality, concentration, purity, and size of APN cmRNAs were evaluated as described in previous reports [31]. In addition, nucleotide analysis was performed. LNP preparation: We employed a robust, self-assembly method to prepare APN mRNA-encapsulated LNPs (Figure 1A) [31,32]. The LNP formulation, LNP preparation methods, LNP characterization, and optimization studies have been extensively characterized and optimized in our previous studies [31]. This formulation has demonstrated effective mRNA delivery in vitro and desirable properties such as for in vivo applications.

### 2.2. Cell Culture and Transfection with APN-LNP

We used 3T3-L1 cells to study adipogenesis due to their established responsiveness to hormonal induction protocols. 3T3-L1 cell line from ATCC (CL-173) was cultured in Dulbecco’s modified Eagle medium with 4.5 g/L D-glucose (DMEM, Gibco, Life Technologies, Carlsbad, CA, USA) supplemented with 10% newborn calf serum (Gibco, Life Technologies) and 1% penicillin-streptomycin (Gibco, Life Technologies). Passages 3-8 were used for the experiment. Cells were seeded on a 6-well plate at a density of 4 × 10^4^ cells/mL and allowed to grow for 2 days to reach 100% cell confluence (D0). Then, cells were induced in Adipogenic Induction medium (AIM), containing 10 μg/mL insulin (Sigma-Aldrich, St. Louis, MO, USA), 1 μM dexamethasome (Sigma-Aldrich), and 0.5 mM isobutylmethylxanthine (IBMX) (Sigma-Aldrich) in complete DMEM medium for 72 h (D1–D3). After that, the medium was changed into Adipogenic Differentiation Medium (ADM), including 10 μg/mL insulin for 48 h (D4–D5). Finally, cells were maintained in DMEM medium for another 2 days (D6–D7). Adipocytes in different differentiation stages were transfected with APN-LNP. In both models, 24 h transfection with APN-LNP (1.0 μg/mL) was performed in Opti-MEM, with preadipocytes treated at differentiation initiation (D0) and mature adipocytes at the fully differentiated stage (D8). Then, cells were harvested for analysis, with an empty-LNP group serving as a control.

The MC3T3-E1 and RAW264.7 cell lines are widely accepted models for osteoblast and osteoclast lineage studies, respectively. The MC3T3-E1 cell line was purchased from ATCC (CRL-2593) and maintained in Alpha MEM (AMEM) (Gibco) supplemented with 10% fetal bovine serum and 1% penicillin/streptomycin. MC3T3-E1 cells were seeded on a 6-well plate at a density of 2 × 10^5^ cells/mL in α-MEM containing 50 μg/mL vitamin C and incubated for 2 days. Transfection was then performed with APN-LNP (0.25 and 1.0 μg/mL) for 24 h in Opti medium. The empty-LNP group served as a control. The RAW264.7 cell line from ATCC (TIB-71) was cultured in complete DMEM. Cells at passages 3–8 were used for the experiments. They were seeded onto a 6-well plate at a density of 2.5 × 10^5^ cells/mL. Transfection was carried out using 0.25 μg/mL APN-LNP for 12 h in Opti-MEM. After transfection, the medium was replaced with AMEM containing 100 μg/mL receptor activator of nuclear factor-kappa B ligand (RANKL) (Pepro Tech Inc., Rocky Hill, NJ, USA) and incubated for 48 h. Cells were collected at 24 and 72 h post-transfection for further analysis.

### 2.3. Cytotoxicity Assays

The 3T3-L1 cells were induced in AIM and in ADM as described above. Transfection with APN-LNP in Opti medium was conducted on D0 and D8. The empty-LNP group was used as a control. Cytotoxicity was evaluated using the Cell Counting Kit-8 (CCK-8, Dojindo, Santa Clara, CA, USA), with absorbance measured at 450 nm.

MC3T3-E1 cells were cultured in α-MEM containing 50 μg/mL vitamin C and incubated for 2 days. Transfection with APN-LNP in Opti medium was conducted. The empty-LNP group was used as a control. Cytotoxicity was evaluated using the CCK-8, as described above.

RAW264.7 cells were cultured in complete DMEM. Transfection with APN-LNP in Opti medium was conducted. The empty-LNP group was used as a control. Cytotoxicity was evaluated using the CCK-8,as described above.

### 2.4. RNA Extraction and qPCR

After transfection, total RNA was extracted with the Quick-RNA Miniprep Kit (R1055). cDNA was synthesized and qPCR was performed with PowerUP SYBR Green Master Mix (Thermo Scientific, Waltham, MA, USA) on a Bio-Rad iQ5 thermal cycler (Bio-Rad Laboratories, Hercules, CA, USA) (Table S1). The fold changes were evaluated using the 2^−ΔΔCt^ method, with GAPDH serving as the internal control.

### 2.5. Western Blot

Total cellular protein was extracted using RIPA Lysis (Santa Cruz Biotechnology, Dallas, TX, USA), and the samples were then denatured at 95 °C for 10 min.

The collected cell culture medium was concentrated using Amicon Ultra −5000 MWCO Centrifugal Filter Devices (Merck Millipore, Darmstadt, Germany) under the manufacturer’s instructions. Briefly, the medium was first centrifuged at 8000 rpm for 5 min to remove cellular debris. Next, 3 mL medium from each group was concentrated to 0.2 mL at 4000 g for 25 min using the Amicon devices. The samples were then resuspended in PBS and concentrated again under the same conditions. This process was repeated three times to fully reconstitute the samples in PBS. Protease inhibitors were added at a ratio of 1:100. The samples were denatured as previously described.

The proteins from cells and culture medium were separated by BoltTM 4–12% Bis-Tris Plus sodium dodecyl sulphate-polyacrylamide gel (SDS-PAGE) (Thermo Fisher Scientific, Waltham, MA, USA) and electrophoretically transferred to PVDF membranes (Merck Millipore, Billerica, MA, USA). Primary antibodies against adiponectin (Millipore, Billerica, MA, USA, AB3269P) (1:1000) and GAPDH (Cell Signaling Technology, Danvers, MA, USA) (1:3000) were used. The bands were visualized with ECL chemiluminescence substrate (Thermo Fisher Scientific). The density of all bands was evaluated using ImageJ software (13.0.6).

### 2.6. Enzyme-Linked Immunosorbent Assay

The secretion of APN protein in the collected cell culture medium was analyzed using an enzyme-linked immunosorbent assay (ELISA) kit (Invitrogen, Carlsbad, CA, USA, KMP0041), following the manufacturer’s protocol.

### 2.7. Femoral Fracture Model in Male Diet-Induced Obese (DIO) Mice

The 17–19-week-old DIO (Jax#380050) male mice were purchased from the Jackson Laboratory (Bar Harbor). A femoral fracture model with a fracture gap size of 0.25 mm was generated using the RISystem Internal Fixation System (RISystem AG, Landquart, Switzerland). APN-LNP was delivered by intravenous injection (IV).

Briefly, ketamine and xylazine (100 mg/kg and 10 mg/kg, respectively) were used to anesthetize the mice. Buprenorphine (1 mg/kg) was administered subcutaneously prior to the surgical procedure. An incision was made along the femur, and the underlying muscle was carefully split along the intermuscular boundary to expose the femur. The initial hole was drilled using the Accu Pen 3V, followed by the insertion of the first screw. The MouseFix Drill- & Saw guide was then positioned on the plate, facilitating the drilling of three additional holes with the Accu Pen 3V. A 0.25 mm full-thickness osteotomy was performed in the mid-femur using a 0.22 mm Gigli saw. Muscles were sutured with 6-0 absorbable sutures, whereas the skin was closed using 6-0 non-absorbable monofilament sutures. On day 3 post-surgery, APN-LNP (10 μg in 120 μL LNP buffer) was administered via intravenous injection. Subsequent IV injections were conducted biweekly; a total of two doses of 10 μg APN-LNP each. Matched empty-LNP or PBS served as controls.

### 2.8. Fasting Weight and Blood Glucose Test

As shown in the experimental timeline, fasting body weight and blood glucose were recorded at each timepoint. Briefly, 4 days before surgery, the mice were fasted for 6 h. Then, fasting body weight and blood glucose were recorded (T0). A total of 2 days after drug administration (T1 and T2), these data were recorded again.

### 2.9. Animal Toxicity Test

For the safety consideration of the APN-LNP, the livers of all mice were collected and processed for histological analysis by H&E staining.

### 2.10. μCT Analysis

The collected femurs were scanned using Bruker’s Skyscan 1172 (Bruker, Kontich, Belgium) at 9.0 μm-voxel resolution at Tufts Medical Center. A 0.5 mm aluminum (Al) X-ray filter was used, and the X-ray settings were 50 kV and 500 μA. Scans were performed in 0.3° rotation steps. Projections were acquired at a nominal resolution of 10 μm, with each slice consisting of 1224 × 820 pixels. Three-dimensional reconstructions were visualized with CTvox 3.3.0 (Bruker, Kontich, Belgium) and analyzed with CTAn 1.19 (Bruker, Kontich, Belgium). The regions of interest were confined to the area between two medial screws, as previously described [33].

### 2.11. H&E and IHC Staining

Following decalcification with 10% EDTA, the femurs were processed into paraffin-embedded sections. Hematoxylin and eosin (H&E) staining and immunohistochemical analysis were conducted as previously described [34]. A primary antibody against adiponectin (Thermo Scientific, Waltham, MA, USA, PA 5145419) was applied at a dilution of 1:200. Digital images were captured using an Olympus DP73 microscope.

### 2.12. RNA Isolation from Organs and qPCR Analysis

Total RNA from organs excluding bone was extracted using TRIzol^TM^ Reagent (Invitrogen, Carlsbad, CA, USA) under the manufacturer’s instructions. For the contralateral femur, RNA was isolated using a combination of TRI Reagent and RNeasy Mini Kit columns (Qiagen, Valencia, CA, USA).

### 2.13. Statistical Analysis

Statistical differences among the experimental groups were evaluated with one-way ANOVA followed by Turkey post hoc analysis. For comparisons between two groups, the unpaired Student’s *t*-test was used. All statistical analyses were performed with Graphpad Prism Version 9.0 (Graphpad software). A *p*-value < 0.05 was considered statistically significant. Results were presented as mean ± SD.

## 3. Results

### 3.1. Cytotoxicity of APN-LNP on 3T3-L1, MC3T3-E1, and Raw264.7 Cell Lines

As APN is one kind of adipokine primarily secreted by adipose tissue, the effect of APN-LNP was first evaluated in the 3T3-L1 cell line. Transfections were performed at two different stages of 3T3-L1 cell differentiation. Cytotoxicity was measured using a CCK-8 assay at various APN-LNP concentrations. The results showed no significant cytotoxic effects at concentrations up to 4 μg/mL in both preadipocytes and mature adipocytes (Figure 1B,C), which were consistent in Raw264.7 cells (Figure S1). In MC3T3-E1 cells, no obvious cytotoxicity was observed at concentrations up to 2 μg/mL (Figure S1), but transfection with 4 μg/mL APN-LNP significantly reduced cell numbers compared to the empty-LNP group. These results indicate that APN-LNP is well-tolerated at concentrations used for gene delivery, supporting its use in regenerative applications.

### 3.2. mRNA and Protein Expression of APN After Transfection with APN-LNP

After establishing the safety profile of APN-LNP, we then evaluated the expression of APN after transfection with APN-LNP by RT-PCR. The results showed significantly increased APN levels in either preadipocytes or mature adipocytes (Figure 1D,E). In preadipocytes, APN mRNA expression was upregulated by more than 11,000-fold compared to the empty-LNP control. In contrast, APN levels in mature adipocytes were approximately 2-fold higher than those in the control group, suggesting that mature adipocytes might be more challenging to transfect effectively. To determine whether protein production of APN was similarly enhanced in mature adipocytes, Western blot analysis was conducted (Figure 1F,G). The results indicated a significant increase in APN protein levels in both cellular extracts (Figure 1H) and cell culture supernatants 24 h post-transfection (Figure 1I).

### 3.3. Transfection with APN-LNP Inhibited the Adipogenesis-Related Gene Expression in 3T3-L1 Cells

Given that transfection with APN-LNP significantly altered APN expression, we further examined whether it also affected other adipogenesis-related gene expression using qPCR. Peroxisome proliferator-activated receptor gamma (PPARγ), a key regulator of adipogenesis, along with lipoprotein lipase (Lpl) and hormone-sensitive lipase (Hsl), showed significantly decreased expression levels in both preadipocytes and mature adipocytes following APN-LNP transfection. These findings indicate that APN-LNP transfection effectively suppressed adipogenesis at different stages of adipocyte differentiation (Figure 1D,E).

Previous studies have reported that APN could promote osteogenesis and inhibit osteoclastogenesis in murine cell lines [19,20]. To further explore the functional impact of APN-LNP transfection, we extended our investigation to MC3T3-E1 pre-osteoblasts and RAW 264.7 macrophage cells.

### 3.4. APN-LNP Transfection Promotes Expression of APN and Osteogenic Markers in MC3T3-E1 Cells

**Figure 2:**
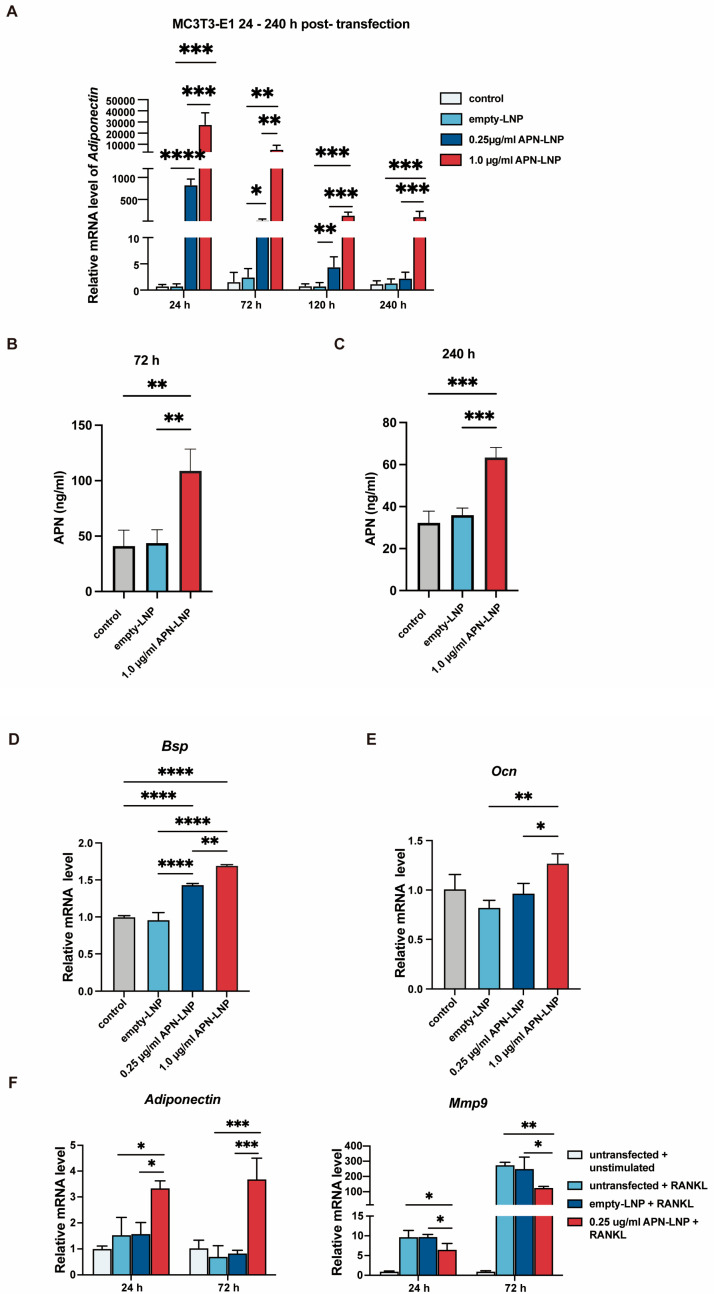
Transfection with APN-LNP promoted osteogenesis in MC3T3-E1 and inhibited *Mmp9* expression in RANKL-stimulated RAW264.7 cells. (**A**) MC3T3-E1 cells were transfected with APN-LNP (0.25 and 1.0 μg/mL) for 24 h, followed by incubation in AMEM. APN mRNA expression was analyzed using qPCR. (**B**,**C**) APN protein levels were measured in the supernatants at 72 and 240 h by ELISA. (**D**,**E**) The expression levels of osteogenesis-related markers, bone sialoprotein (*Bsp*), and osteocalcin (*Ocn*), were also evaluated. (**F**) RAW 264.7 cells were transfected with APN-LNP at 0.25 μg/mL for 12 h and subsequently stimulated with 100 μg/mL RANKL in AMEM. Empty-LNP served as a control. APN and *Mmp9* gene expression were assessed separately using qPCR. * *p* < 0.05, ** *p* < 0.01, *** *p* < 0.001 and **** *p* < 0.0001. Data were expressed as the mean ± SD at three biological independent experiments.

We transfected MC3T3-E1 cells using two concentrations of APN-LNP: 0.25 and 1.0 μg/mL. The transfected cells exhibited a significant, dose-dependent increase in APN mRNA levels at 24, 72, and 120 h after transfection (Figure 2B). Peak expression was observed at 24 h, with the 1.0 μg/mL group showing an over 28,000-fold increase compared to the empty-LNP control, and the 0.25 μg/mL group showing an increase of more than 800-fold. Elevated mRNA levels were sustained for up to 120 h in the 0.25 μg/mL group and up to 240 h in the 1.0 μg/mL group, demonstrating the long-lasting effects of APN-LNP transfection.

Consistent with the mRNA expression data, ELISA analysis at 72 h and 240 h post-transfection demonstrated a significant increase in APN secretion in the 1.0 μg/mL APN-LNP group compared to the control and empty-LNP groups (Figure 2C). APN secretion remained significantly elevated in the 1.0 μg/mL group at 240 h, indicating sustained protein production over time. These results confirm that APN-LNP transfection in MC3T3-E1 cells effectively enhances APN expression at the mRNA and protein levels.

In addition, transfection with APN-LNP in MC3T3-E1 cells promoted the expression of bone sialoprotein (*Bsp*) and osteocalcin (*Ocn*) dose-dependently (Figure 2D,E). Notably, the highest *Bsp* and *Ocn* mRNA levels were observed in the 1.0 μg/mL APN-LNP group.

### 3.5. Transfection of RAW 264.7 Cells with APN-LNP Reduces Mmp9 Expression

RAW 264.7 cells were transfected with APN-LNP, and the expression of matrix metalloproteinase 9 (Mmp9) was evaluated. The results indicated that APN-LNP decreased *Mmp9* expression at 24 and 72 h post-transfection (Figure 2F).

### 3.6. Fasting Bone Healing and Metabolic Response to APN-LNP Treatment of Femoral Fracture Mice

**Figure 3:**
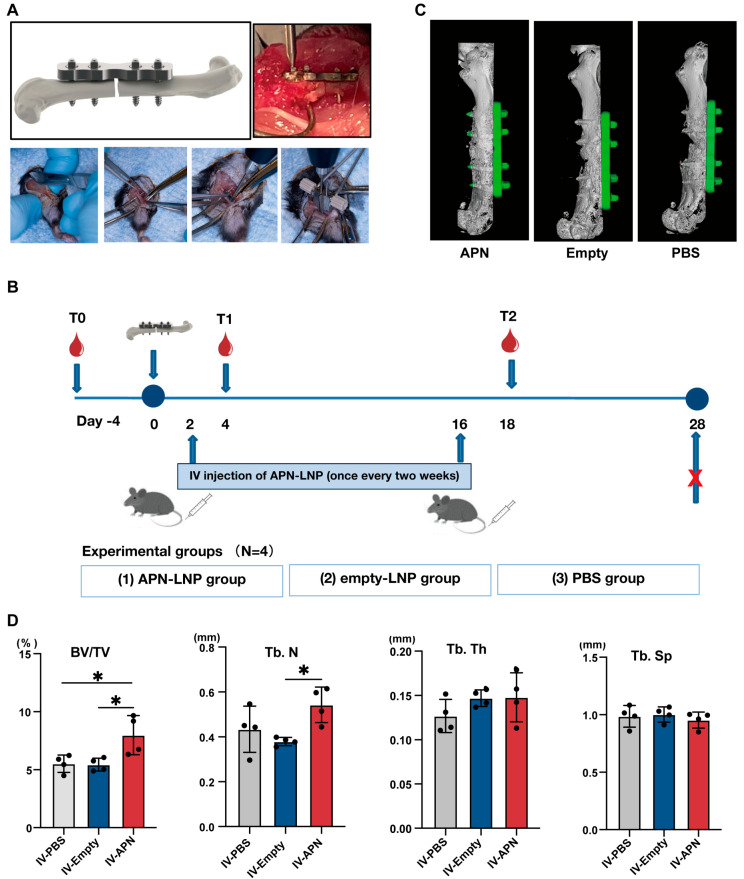
Schematic representation of (**A**) surgical procedure of 0.25 mm defect using RISystem Internal Fixation System and (**B**) surgery timeline of the femoral fracture model in male DIO mice. (**C**) 3D images of the three groups at 4 weeks post-surgery. (**D**) The results of BV/TV (%), Tb. N, Tb. Th, and Tb. Sp. * *p* < 0.05. N = 4.

Based on these in vitro findings, we next investigated the efficacy of APN-LNP in vivo. We established a femoral fracture model with a 0.25 mm fracture gap using the RISystem Internal Fixation System in male DIO mice (Figure 3A). Systemic IV injection of APN-LNP was performed on the third day after surgery (Figure 3B). Totally, two doses of APN-LNP were administered. The second dose was given two weeks after the first administration.

**Figure 4:**
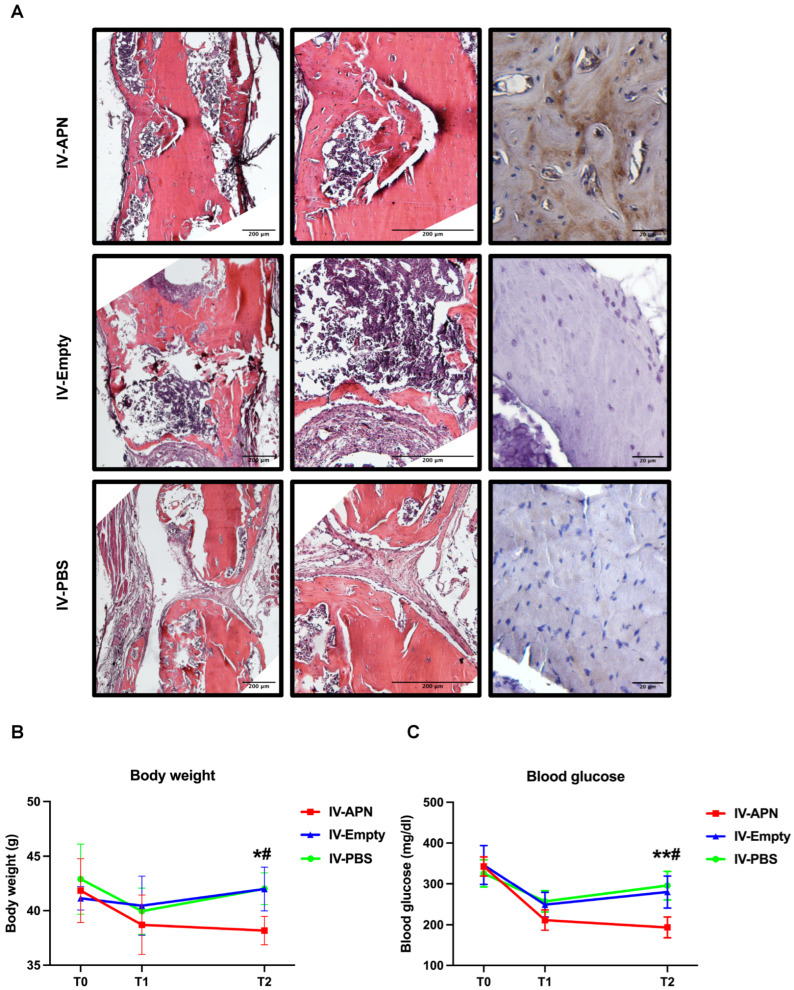
Histopathology results of fractured femurs, body weight, and blood glucose in APN-LNP-treated DIO mice. (**A**) H&E and immunohistochemistry stainings of femoral defects 4 weeks after surgery. Fasting blood glucose (**B**) and body weight (**C**) of the experimental mice at different timepoints. * vs. PBS-LNP group, # VS. Empty-LNP group. */# *p* < 0.05 and ** *p* < 0.01. N = 4.

At 4 weeks after surgery, we evaluated bone healing in the fracture site. Micro-CT analysis revealed that the bone volume to total volume ratio (BV/TV) and trabecular number (Tb.N) were significantly higher in the APN-LNP group compared to the empty-LNP and PBS groups, whereas no obvious differences was found among the three groups in regarding to trabecular thickness (Tb.Th) and trabecular separation (Tb.Sp) (Figure 3C,D). H&E staining demonstrated bone healing at the femoral fracture site (Figure 4A). In situ expression of APN was confirmed through immunohistochemical staining (Figure 4A).

To evaluate whether the administration of APN-LNP could influence fasting blood glucose and body weight, timepoints T1 and T2 were set up two days after each administration of APN-LNP. At baseline, there was no significant difference among the three experimental groups in body weight or fasting blood glucose. At T1, 2 days after the first dose of administration, blood glucose and body weight decreased in all groups. At T2, 2 days after the second dose of administration, blood glucose and body weight in the IV-APN group significantly decreased, compared with the IV-empty and IV-PBS groups (Figure 4B,C).

### 3.7. H&E and Immunohistochemistry Staining of Liver Tissues

**Figure 5:**
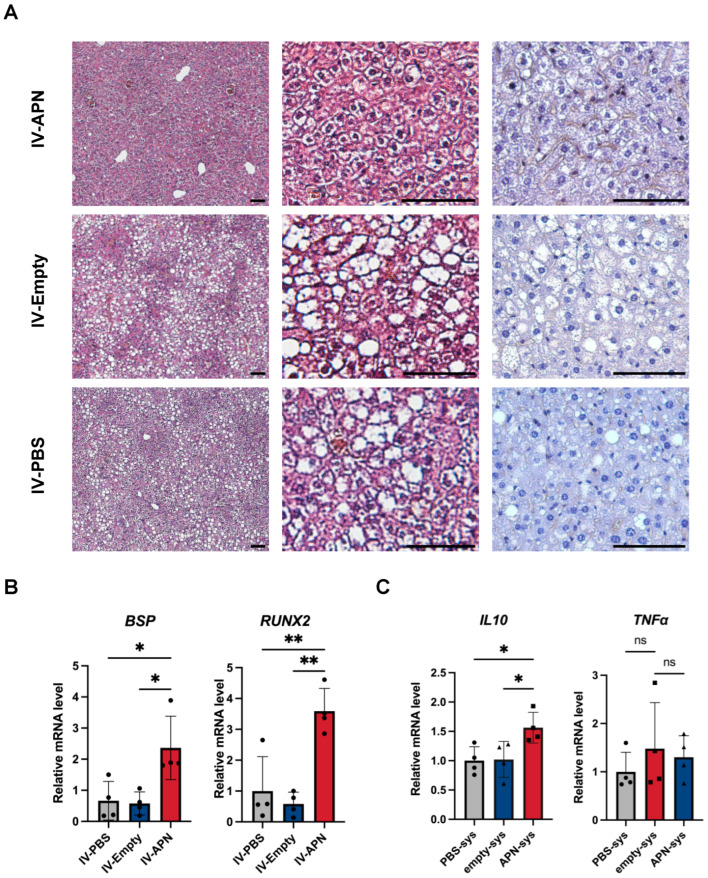
Histopathology results of liver tissues and gene expression of some markers in different organs of experimental groups 4 weeks after surgery. (**A**) H&E and immunohistochemistry staining for adiponectin of liver tissues. Scale bars = 50 μm. (**B**) Gene expression of osteogenic markers *Bsp* and *Runx2* in contralateral femur tissues. (**C**) Gene expression of anti-inflammatory markers *IL-10*, as well as proinflammatory markers *TNFα*, in WAT. * *p* < 0.05 and ** *p* < 0.01. ns = not significant. N = 4.

We also studied the systemic toxicity of APN-LNP and empty-LNP through histological analysis of the liver. H&E staining of liver tissues revealed no significant damage across all groups (Figure 5A). Additionally, APN expression was confirmed by immunohistochemical staining (Figure 5A).

### 3.8. Osteogenic Markers Bsp and Runx2 Expression in Contralateral Femurs of the Experimental Mice

Analysis of contralateral femur tissues revealed that mRNA expression levels of osteogenic markers *Bsp* and *Runx2* were significantly elevated in the APN-LNP group compared to both the empty-LNP and control groups (Figure 5B).

### 3.9. Inflammation Related Markers TNFα and IL-10 in White Adipose Tissues (WAT)

Additionally, we assessed the influence of APN-LNP on the expression of inflammatory cytokines in WAT. We found that APN-LNP administration increased the mRNA levels of anti-inflammatory marker *IL-10* (Figure 5C). Nevertheless, no differences were found regarding gene expression of pro-inflammatory marker *TNFα* (Figure 5C).

## 4. Discussion

During the past decades, gene therapy has been rapidly developed [27]. Since COVID-19, the application of mRNA therapy delivered by LNPs has triggered researchers’ interests and is being studied deeply, especially in treating cancer and infectious diseases [22,23,24,25,26,35]. Metabolic diseases, such as diabetes and obesity, have become major health problems worldwide. Although traditional treatment methods are effective in general, there are still some limitations. APN is a very important adipokine. It functions through improving insulin resistance, regulating lipid metabolism, promoting osteogenesis, and inhibiting osteoclastogenesis [18,19,20,36]. Despite the promising usage of protein APN, its clinical application is hindered by some disadvantages, including the high dosage required in treatment, different isoforms of APN, and constant IV injection to obtain beneficial effects. Our studies modified the APN messenger RNA and packaged the mRNA with LNPs to form the APN-LNP. Compared to the recombinant APN protein, which has a short half-life and requires frequent injections, mRNA-LNPs enable sustained protein expression in vivo. Unlike viral vectors, LNPs avoid genomic integration, reducing oncogenic risk. These properties position mRNA-LNPs as a safer, scalable alternative for therapeutic delivery [21,24]. We aimed to explore the effects of this APN-LNP on adipogenesis and bone metabolism in vitro and in vivo.

Adiponectin (APN) is primarily secreted by adipose tissue. In this study, we demonstrated that APN-LNP successfully transfected 3T3-L1 adipocytes, leading to an upregulation of APN gene and protein expression in this cell line. Furthermore, elevated APN protein levels were detected in both cellular extracts and the cell culture supernatant of transfected mature adipocytes, indicating that the modified APN-LNP mRNA delivered by lipid nanoparticles (LNPs) can be translated into protein transcripts in vitro as anticipated. We also found that APN-LNP inhibited the expression of adipogenesis-related markers, such as *Pparγ*, *Lpl*, and *Hsl*, during two stages of adipocyte differentiation. Several studies have identified *Pparγ* as a critical regulator of adipogenesis and lipid accumulation [37,38,39]. Overall, our data suggest that transfection with APN-LNP enhances the gene and protein expression of APN, subsequently leading to decreased expression of adipogenic genes, indicating a potential anti-adipogenic effect in 3T3-L1 cells. Supporting our findings, one study reported that overexpression of APN suppressed adipogenesis in cultured bone marrow mesenchymal stem cells [40].

APN is expressed in adipocytes, as well as in human and murine osteoblasts and myocytes [41,42]. To evaluate the APN-LNP transfection effect on bone metabolism, we used the MC3T3-E1 osteoblastic cell line and the RAW264.7 cell line, the latter of which can be differentiated into osteoclasts upon RANKL stimulation and is frequently used for osteoclastic research. These cell lines are commonly utilized in in vitro studies of bone metabolism. Our results indicate that transfection with APN-LNP in stimulated MC3T3-E1 cells upregulated APN expression at both the mRNA and protein levels over several days. Notably, as the concentration of APN-LNP increased, this effect persisted for a longer duration. Additionally, we demonstrated that APN-LNP transfection in MC3T3-E1 osteoblastic cells upregulated the expression of *Bsp* and *Ocn* dose dependently, suggesting that APN-LNP may promote osteogenesis. Other researchers have reported that APN treatment enhances cell proliferation and the expression of osteogenic markers in osteoblasts derived from both murine and human sources [19,20].

Regarding the effect of APN on osteoclasts, various studies reported a negative role of APN on osteoclastogenesis in RAW264.7 cells [18,19,20]. Our results demonstrated that transfection with APN-LNP significantly reduced *Mmp9* expression levels in RANKL-stimulated RAW264.7 cells. Elevated *Mmp9* levels produced by osteoclasts are crucial for extracellular matrix degradation and osteoclastic bone resorption [43,44]. Thus, our findings suggest that APN-LNP may inhibit osteoclastogenesis by downregulating *Mmp9* expression. While previous research has indicated that APN can inhibit osteoclastogenesis, this study presents the first evidence of its inhibitory effect on *Mmp9* production in RAW264.7 cells. Further studies will be performed to investigate the underlying mechanisms.

In this study, we aimed to explore the impact of APN-LNP administration on bone healing and metabolic parameters in a femoral fracture model of diet-induced obesity (DIO) mice. At timepoint T1, we observed a decrease in fasting blood glucose levels and body weight not only in the APN-LNP group but also in both the empty-LNP and control groups, likely due to the influence of the femoral surgery. DIO mice are known to be sensitive to environmental factors and interventions, and the surgical procedure may have significantly impacted their physiological status. At timepoint T2, however, the APN-LNP group exhibited a significant reduction in blood glucose and body weight, indicating a beneficial effect of APN-LNP on metabolic regulation.

Numerous in vitro studies have confirmed adiponectin (APN)’s positive effects on osteogenesis, which support our findings [18,19,20]. In this study, we demonstrated that APN-LNP treatment partially enhances bone healing, as evidenced by a higher BV/TV ratio in APN-LNP-treated mice compared to those treated with empty-LNP and PBS. Furthermore, analysis of contralateral femur tissues revealed significantly elevated mRNA expression levels of osteogenic markers *Bsp* and *Runx2* in the APN-LNP group relative to both the empty-LNP and control groups. Additionally, immunohistochemistry results confirmed positive APN expression in the APN-LNP group. These data suggest that the overexpression of APN promotes bone healing at the fracture sites in DIO mice, possibly through the upregulation of osteogenic markers such as *Bsp* and *Runx2*.

It is important to consider that the physiological environment is highly complex, and the bone healing process can be influenced by various factors, including fracture type, patient age, health status, and underlying medical conditions. Therefore, while APN-LNP may effectively promote bone healing in specific contexts, its efficacy may not be universally applicable across all fracture types.

This study provides preliminary evidence for the therapeutic potential of APN-LNP in bone healing and metabolic regulation, while highlighting several important directions for future investigation. First, the short study duration and use of only male DIO mice in our study limit generalizability. Longitudinal studies incorporating multiple time points and evaluating sex differences are needed to fully characterize the temporal effects of APN on bone metabolism. Second, while our current study provides valuable transcriptomic insights, functional validation of APN-LNP’s effects across different cell types, such as Oil Red O staining for adipogenesis, Alizarin Red staining for osteoblast mineralization, and TRAP staining for osteoclastogenesis, would significantly enhance our understanding of APN-LNP’s cellular mechanisms. Third, comprehensive mechanistic studies should explore both the anti-inflammatory properties of APN-LNP and its interactions with obesity-related metabolic pathways. Although H&E staining revealed no apparent hepatic toxicity, systematic dose-response studies coupled with specific biochemical markers and pharmacokinetic analyses will be essential for clinical translation. Finally, advanced molecular techniques, such as RNA sequencing and pathway analysis, could provide valuable insights into the precise mechanisms underlying APN-LNP’s therapeutic effects.

Our previous work demonstrated that APN-LNP improved insulin sensitivity and reduced inflammation in diabetic kidneys [32]. Here, we show similar anti-inflammatory and osteogenic effects in bone. Specifically, both studies revealed the ability of APN-LNP to reduce inflammation, suggesting a common underlying mechanism of action. While the previous study focused on metabolic improvements, the current investigation highlights the treatment of bone disorders. Together, these results support APN-LNP’s potential as a multi-system therapy for metabolic and skeletal complications of obesity, warranting further exploration of its mechanisms of action.

## 5. Conclusions

**Figure 6:**
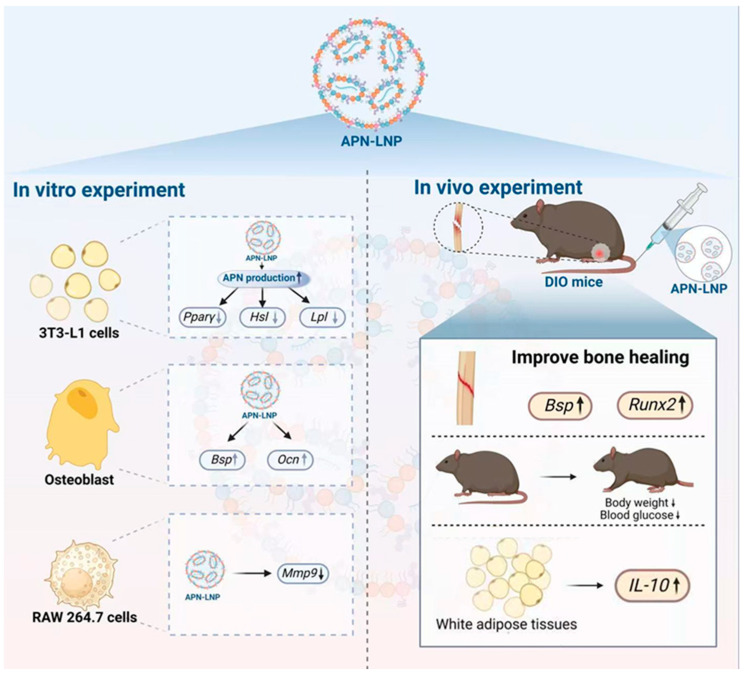
Schematic overview of the actions of APN-LNP. Transfection with APN-LNP inhibited adipogenesis in 3T3-L1 adipocytes and reduced *Mmp9* expression in RANKL-stimulated RAW264.7 cells. Additionally, APN-LNP enhanced osteogenesis in MC3T3-E1 cells. In vivo studies demonstrated that intravenous injection of APN-LNP promoted bone healing in a femoral fracture model of diet-induced obesity (DIO) mice. Furthermore, administration of APN-LNP led to decreased fasting blood glucose and body weight and increased *IL-10* expression in white adipose tissue, thereby improving obesity, metabolic function, and inflammation in the DIO mice.

In conclusion, our results suggest that APN-LNP could inhibit adipogenesis and promote osteogenesis. Moreover, we found that APN-LNP improved bone healing by promoting osteogenesis and reducing inflammation in a femoral fracture model of male DIO mice. Overall, this study provides promising preclinical data on the potential of APN-LNP as a novel therapy for obesity-related bone disorders, offering both metabolic and osteogenic benefits. (Figure 6).

## Data Availability

All data that support the findings of this study are available from the corresponding authors upon reasonable request.

## Supplementary Materials

The following supporting information can be downloaded at: https://www.mdpi.com/article/10.3390/cells14120891/s1, Figure S1: Cytotoxicity of APN-LNP in MC3T3-E1 and RAW 264.7 cell lines; Table S1: Primer sequences used in qPCR experiments.
