# Supplementary material for: Effects of Modified Messenger RNA of Adiponectin Delivered by Lipid Nanoparticles on Adipogenesis and Bone Metabolism In Vitro and In Vivo
Source: Cells. 2025 Jun 13;14(12):891. doi: 10.3390/cells14120891 (PMC12190357; doi:10.3390/cells14120891)
Supplement: Supplementary file 1 [file cells-14-00891-s001.zip › Figure S1.pdf]

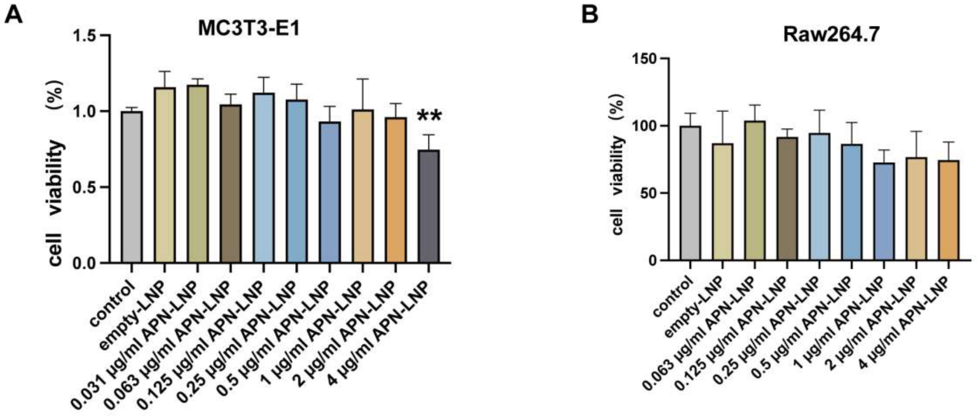


**Figure S1.** Evaluation of APN-LNP cytotoxicity in MC3T3-E1 and RAW 264.7 cell lines. (**A**) MC3T3-E1 cells were cultured in α-MEM containing 50 μg/ml vitamin C and incubated for 2 days. Transfection with APN-LNP in Opti medium was conducted. The empty-LNP group was used as a control. Cytotoxicity was evaluated using the CCK-8 kit. (**B**) RAW 264.7 cells were transfected with APN-LNP in Opti medium for 12 h. The empty-LNP group was used as a control. Cytotoxicity was evaluated using the CCK-8 kit. Data are expressed as mean ± SD at three biological independent experiments. **$p < 0.01$.
