# Supplementary material for: Effects of Modified Messenger RNA of Adiponectin Delivered by Lipid Nanoparticles on Adipogenesis and Bone Metabolism In Vitro and In Vivo
Source: Cells. 2025 Jun 13;14(12):891. doi: 10.3390/cells14120891 (PMC12190357; doi:10.3390/cells14120891)
Supplement: Supplementary file 1 [file cells-14-00891-s001.zip › Table S1.pdf]

Table S1. Primer sequences used in qRT-PCR experiments.

Gene	Forward primer (5' - 3')	Reverse primer (5'- 3')
Adiponectin	GCACTGGCAAGTTCTACTGCAA	GTAGGTGAAGAGAACGGCCTTGT
Pparγ	GAAAGACAACGGACAAATCACC	GGGGGTGATATGTTTGAACTTG
Lpl	GGACGGTAACGGGAATGTATGA	TGACATTGGAGTCAGGTTCTCTCT
Hsl	AGACCACATCGCCCACA	CCTTTATTGTCAGCTTCTTCAAGG
Bsp	CAGGGAGGCAGTGACTCTTC	AGTGTGGAAAGTGTGGCGTT
Ocn	GCCGGAGTCTGCTCACTACC	GCGCTCTGTCTCTCTGACCT
Mmp9	GCAGAGGCATACTTGTACCG	TGATGTTATGATGGTCCCACTTG
IL-10	GCTCTTACTGACTGGCATGAG	CGCAGCTCTAGGAGCATGTG
TNF-α	TGTCCCTTTCACTCACTGGC	CATCTTTTGGGGGAGTGCCT
Gapdh	AGGTCGGTGTGAACGGATTTG	TGTAGACCATGTAGTTGAGGTCA
